# Parental History and Lifestyle Behaviors in Relation to Mortality From Stroke Among Japanese Men and Women: The Japan Collaborative Cohort Study

**DOI:** 10.2188/jea.JE20110163

**Published:** 2012-07-05

**Authors:** Eri Eguchi, Hiroyasu Iso, Yasuhiko Wada, Shogo Kikuchi, Yoshiyuki Watanabe, Akiko Tamakoshi

**Affiliations:** 1 Public Health, Department of Social and Environmental Medicine, Graduate School of Medicine, Osaka University, Suita, Osaka, Japan; 2 Department of Nutrition, University of Kochi, Kochi, Japan; 3 Department of Public Health, Aichi Medical University School of Medicine, Nagakute, Aichi, Japan; 4 Department of Epidemiology for Community Health and Medicine, Kyoto Prefectural University of Medicine Graduate School of Medical Sciences, Kyoto, Japan

**Keywords:** parental history, stroke, lifestyle, cohort study

## Abstract

**Background:**

We assessed the impact of parental history of stroke on stroke mortality, as well as the effect modification between lifestyle and stroke mortality, among Japanese.

**Methods:**

In this community-based, prospective cohort study, 22 763 men and 30 928 women aged 40 to 79 years with no history of cardiovascular disease or cancer at baseline (1988–1990) were followed through 2008. We examined the association between parental history of stroke and stroke mortality and estimated the impact of the combination of lifestyle and parental history on stroke mortality in offspring.

**Results:**

During a mean follow-up period of 15.9 years, there were 1502 stroke deaths. In both sexes, participants with a parental history of stroke had a higher risk of stroke mortality as compared with those without such a history. The respective multivariable hazard ratio (95% CI) and population attributable fraction were 1.28 (1.10–1.49) and 5.4% in men, 1.22 (1.04–1.43) and 4.3% in women, and 1.25 (1.12–1.40) and 4.8% in all participants, for offspring with a maternal and/or paternal history of stroke. There was an inverse association between healthy-lifestyle score and stroke mortality, irrespective of parental history of stroke. The overall multivariable hazard ratio for the highest (6–8) versus the lowest (0–3) score categories was 0.56 (95% CI, 0.43–0.72) for participants with a maternal and/or paternal history of stroke and 0.44 (0.36–0.53) for those without such a history.

**Conclusions:**

Parental history of stroke was associated with stroke mortality in offspring. The inverse association between healthy lifestyle behaviors and stroke mortality, regardless of parental history, suggests that lifestyle modification is beneficial, even among individuals with a parental history of stroke.

## INTRODUCTION

There is substantial evidence of a relationship between a family history of coronary heart disease in parents and increased risk of coronary heart disease in their offspring.^1^^,^^2^ However, the evidence of such an association with stroke is limited, and the results of studies have been inconsistent.^3^^–^^5^ Current guidelines for assessment of cardiovascular risk^6^ recommend consideration of a parental history of early-onset cardiovascular disease (onset age <55 years in fathers or <65 years in mothers) as a risk factor for incident cardiovascular disease in their offspring.^6^^,^^7^

Well-established risk factors for stroke include hypertension, diabetes mellitus, and obesity, which are largely hereditary.^8^^–^^12^ Although lifestyle behaviors such as diet, exercise, and smoking can also be hereditary, they are largely influenced by family situations in which both parents and offspring exhibit similar lifestyle behaviors. We previously reported an inverse association between the extent of healthy lifestyle behaviors and mortality from stroke, coronary heart disease, and total cardiovascular disease in the general Japanese population.^13^ It is uncertain, however, whether this association is modified by parental history of stroke. Hence, knowledge of an association between healthy lifestyle behaviors and stroke mortality, stratified by parental history of stroke, would be important for motivating individuals with a parental history of stroke to improve their lifestyle behaviors and reduce their stroke risk.

In this large, prospective cohort study of samples of Japanese men and women from the general population, we examined the association of parental history of stroke with stroke-related mortality risk in their offspring and determined whether the association between lifestyle behaviors and stroke mortality was modified by parental history.

## METHODS

### Study population

The Japan Collaborative Cohort Study (JACC Study) was conducted between 1988 and 1990 and enrolled adults living in 45 areas of Japan. The sampling methods and details of the JACC study are described elsewhere.^13^^–^^17^ Participants were recruited mainly at the time of health check-ups, using a self-administered questionnaire that asked about lifestyle behaviors and parental medical history of stroke. The response rate to the questionnaire was 83%. We followed 110 792 adults (46 465 men and 64 327 women) aged 40 to 79 years at baseline. Of these individuals, 26 826 (11 121 men and 15 705 women) were excluded due to the absence of information on parental history of cardiovascular disease, 3837 (1747 men and 2090 women) were excluded due to a positive history of stroke, coronary heart disease, or cancer, and 30 275 (12 581 men and 17 694 women) were excluded due to the absence of information necessary for calculating healthy-lifestyle scores. Data from 53 691 (22 763 men and 30 928 women) were ultimately included in the analysis. There were no important differences between individuals who had complete information on parental history and those who did hot.

### Mortality surveillance

Date and cause of death were confirmed by reviewing all death certificates in each study area, with the permission of the Director-General of the Prime Minister’s Office, up to the end of 31 December 2008, except for 11 areas in which follow-up was terminated at the end of 1999 (4 areas) or 2003 (4 areas). Stroke mortality was determined according to the *International Classification of Diseases*, 10th revision (I60 to I69). A total of 11 099 subjects were censored because of death, and 2863 subjects were censored due to departure or exclusion from the study community. The mean follow-up period was 15.9 years. The study design was approved by the Ethical Board of Nagoya University School of Medicine, the University of Tsukuba, and Osaka University.

### Statistical analysis

Parental history of stroke was classified as maternal, paternal, or maternal and/or paternal. We first calculated sex-specific mean age, age-adjusted mean healthy-lifestyle score, and prevalence of stroke risk factors for participants with and without a paternal and/or maternal history of stroke using analysis of covariance for mean values and a logistic regression model for prevalence. We used Cox proportional hazards models to calculate hazard ratios with 95% CIs to determine sex-specific age-adjusted and multivariable-adjusted associations between parental history and stroke mortality associated with non-maternal, non-paternal, and no history of paternal stroke. We constructed 2 multivariable hazard models. The covariates for model 1 were baseline age (in years), healthy-lifestyle score (discussed below; 0–3, 4, 5, 6–8 points), perceived mental stress (low, moderate, high), level of education (<13, 13–15, 16–18, ≥19 years), regular employment (yes, no), and sex (in the analysis of all participants). Covariates for model 2 were those for model 1 plus hypertension and history of diabetes mellitus. Population attributable fractions (PAFs) were calculated with the standard method,^18^ using multivariable hazard ratios (model 1) with adjustment for history of hypertension and history of diabetes mellitus, as these histories are regarded as contributory intermediate factors in the causal relationship. To examine whether the magnitudes of associations between parental history and stroke mortality differed with regard to maternal or paternal history of stroke, we used an interaction term for maternal and paternal histories. We then repeated the analysis of all participants after stratification by age category (40–59 vs 60–79 years).

In addition, we calculated sex-specific multivariable hazard ratios and 95% CIs for stroke mortality for each healthy-lifestyle score category—with the worst score category of 0 to 3 points as the reference category—stratified by parental history of stroke status for the comparison of stroke mortality with regard to family history of stroke. We previously reported the details of the components of the healthy-lifestyle score.^13^ In summary, participants scored 1 point for each of the following healthy lifestyle behaviors: consumption of at least 1 serving of fruit per day, at least 1 serving of fish per day, regular consumption of milk, habitual exercise or walking (exercise ≥5 hours per week and/or walking ≥1 hour per day), body mass index (BMI) of 21 to 25 kg/m^2^, ethanol intake less than 46.0 g per day, no smoking habit, and sleep duration of 5.5 to 7.4 hours per day. Total score ranged from 0 to 8, and scores were grouped into 4 categories (0–3, 4, 5, 6–8 points) for analysis. We combined scores of 0 through 3 and those of 6 through 8 to keep the number of participants roughly balanced in each category. Covariates for multivariable analyses were baseline age (in years), perceived mental stress (low, moderate, high), level of education (<13, 13–15, 16–18, ≥19 years), and regular employment (yes, no). We again repeated the analysis of all participants after stratification by age category (40–59 vs 60–79). To illustrate the magnitude of associations between healthy lifestyle and stroke mortality between participants with and without a parental history of stroke, we repeated the analysis after stratification by parental history of stroke, with the category of worst lifestyle score (0–3) plus positive parental history of stroke as the reference category. Seven HRs were calculated as 3 score categories with a positive family history and 4 score categories with a negative family history. We used the statistical analysis software (SAS) program Ver. 9.13 (SAS Institute Inc., Cary, NC, USA) for all statistical analyses. All probability values for statistical tests were 2-tailed, and values of *P* less than 0.05 were regarded as statistically significant.

## RESULTS

The mean age of participants was 56.5 years in men and 56.8 years in women, mean BMI was 22.7 in men and 22.9 in women, and a history of stroke in 1 or both parents was noted in 24.6% of men and 23.9% of women. During 855 758 person-years of follow-up (353 588 in men and 502 170 in women), there were 1502 deaths due to stroke (774 in men and 728 in women).

Table 1 shows sex-specific mean age, age-adjusted mean healthy-lifestyle scores, and prevalences of stroke risk factors at baseline among participants with and without a maternal and/or paternal history of stroke. As compared with participants without a parental history of stroke, both men and women with a parental history of stroke were likelier to be older, have a higher healthy-lifestyle score, and have a history of hypertension. They were also likelier to consume milk on an almost daily basis and to habitually exercise or walk. Men were likelier to be nonsmokers and heavy drinkers, and women were likelier to consume at least 1 serving of fish per day and were less likely to have graduated from college or university.

**Table 1.** Mean age, age-adjusted mean healthy-lifestyle score, and prevalences of stroke risk factors in subjects with and without a parental history of stroke

			Maternal and/or paternal history of stroke		*P*-value
			
			−	+	
**Men**					
	No. at risk		17 170	5593	
		Age, years	56.1	58.0	<0.001
		Healthy-lifestyle score, points	3.95	4.00	0.05
		Fruit ≥1/day^a^, %	46.2	46.2	1.00
		Fish ≥1/day^a^, %	30.6	31.6	0.16
		Milk almost daily^a^, %	38.6	41.0	0.002
		Habitual exercise or walking^a^, %	65.4	67.2	0.01
		BMI 21–25 kg/m^2 a^, %	51.3	52.2	0.20
		Ethanol intake <46.0 g/day^a^, %	69.9	66.8	<0.001
		Nonsmoker^a^, %	46.3	48.6	0.003
		Sleep 5.5–7.4 hours/day^a^, %	46.8	46.0	0.33
		High perceived mental stress, %	23.5	24	0.44
		History of hypertension, %	18.0	26.1	<0.001
		History of diabetes mellitus, %	5.8	5.8	0.92
		College or higher education, %	18.1	18.5	0.45
		Regular employment, %	75.7	76.5	0.20
**Women**					
	No. at risk		23 541	7387	
		Age, years	56.3	58.4	<0.001
		Healthy-lifestyle score, points	5.06	5.13	<0.001
		Fruit ≥1/day^a^, %	60.7	61.2	0.39
		Fish ≥1/day^a^, %	32.0	35.0	<0.001
		Milk almost daily^a^, %	44.9	45.6	0.31
		Habitual exercise or walking^a^, %	66.7	67.2	0.40
		BMI 21–25 kg/m^2 a^, %	47.5	48.4	0.21
		Ethanol intake <46.0 g/day^a^, %	99.3	99.3	0.92
		Nonsmoker^a^, %	95.6	96.1	0.09
		Sleep 5.5–7.4 hours/day^a^, %	59.7	59.9	0.83
		High perceived mental stress, %	20.1	20.8	0.21
		History of hypertension, %	19.4	27.6	<0.001
		History of diabetes mellitus, %	3.2	3.5	0.26
		College or higher education, %	10.8	9.4	0.001
		Regular employment, %	33.6	34.5	0.20

^a^Component of healthy-lifestyle score (0–8).

Table 2 shows sex-specific age-adjusted and multivariable hazard ratios with 95% CIs and PAFs for mortality from stroke by parental history of stroke. Both men and women with a parental history of stroke had a higher age-adjusted risk of stroke mortality than did those without a parental history of stroke. Hazard ratios tended to be higher among those with a maternal versus a paternal stroke history; however, this difference was not statistically significant (*P* = 0.49). In model 1, the overall multivariable hazard ratio (95% CI) was 1.33 (1.16–1.52) for maternal stroke history, 1.16 (1.03–1.32) for paternal stroke history, and 1.25 (1.12–1.40) for maternal and/or paternal history of stroke. The hazard ratios were lower in model 2, which was further adjusted for history of hypertension and diabetes mellitus.

**Table 2.** Sex-specific age-adjusted and multivariable hazard ratios (HRs), 95% CIs, and population attributable fractions (PAFs) for mortality from stroke, stratified by parental history of stroke

			Men	Women	Total
**Person-years**			353 588	502 170	855 758
	**Positive maternal history**				
		No.	130	125	255
		Age-adjusted HR (95% CI)	1.24 (1.03–1.50)	1.37 (1.13–1.66)	1.31 (1.15–1.50)
		Multivariable HR1 (95% CI)^a^	1.26 (1.05–1.53)	1.40 (1.15–1.70)	1.33 (1.16–1.52)
		Multivariable HR2 (95% CI)^b^	1.17 (0.97–1.42)	1.28 (1.06–1.56)	1.21 (1.06–1.39)
		PAF (%)^c^	2.4	3.2	2.8
	**Positive paternal history**				
		No.	158	148	306
		Age-adjusted HR (95% CI)	1.16 (0.97–1.38)	1.17 (0.98–1.40)	1.17 (1.03–1.32)
		Multivariable HR1 (95% CI)^a^	1.17 (0.99–1.40)	1.15 (0.96–1.37)	1.16 (1.03–1.32)
		Multivariable HR2 (95% CI)^b^	1.09 (0.91–1.30)	1.08 (0.90–1.30)	1.08 (0.95–1.23)
		PAF (%)^c^	2.5	2.1	2.3
	**Positive maternal and/or paternal history**				
		No.	244	221	465
		Age-adjusted HR (95% CI)	1.24 (1.07–1.45)	1.22 (1.04–1.43)	1.24 (1.11–1.38)
		Multivariable HR1 (95% CI)^a^	1.28 (1.10–1.49)	1.22 (1.04–1.43)	1.25 (1.12–1.40)
		Multivariable HR2 (95% CI)^b^	1.18 (1.02–1.38)	1.13 (0.97–1.33)	1.16 (1.04–1.29)
		PAF (%)^c^	5.4	4.3	4.8

^a^Multivariable HR1: adjusted for age, sex (for all subjects), healthy-lifestyle score, perceived mental stress, educational level, and regular employment.

^b^Multivariable HR2: adjusted for the above variables plus history of hypertension and history of diabetes mellitus.

^c^PAFs were calculated with the multivariable HR1.

The overall PAF for stroke mortality, 4.8%, was higher among participants with a maternal and/or paternal history of stroke than among those with only a maternal or only a paternal history.

We analyzed parental history and stroke mortality stratified by age category (not shown in table). Participants aged 40 to 59 years had higher age-adjusted and multivariable hazard ratios of stroke mortality for both maternal and paternal history of stroke than did those aged 60 to 79 years. In model 1, the respective multivariable hazard ratios (age 40–59 years vs 60–79 years) were 1.47 (95% CI, 1.05–2.04) versus 1.29 (1.11–1.50) for positive maternal history, 1.65 (1.25–2.18) versus 1.07 (0.93–1.24) for positive paternal history, and 1.63 (1.26–2.09) versus 1.18 (1.04–1.33) for positive maternal and/or paternal history of stroke. The respective PAFs for mortality from stroke were 3.0% versus 3.2% for positive maternal history, 6.0% versus 1.2% for positive paternal history, and 8.3% versus 4.3% for positive maternal and/or parental history.

Table 3 shows multivariable hazard ratios of mortality from stroke for each healthy-lifestyle score category, stratified by parental history of stroke with the 0 to 3 score (worst score) category as the reference. Overall, the risk of stroke mortality decreased with an increase in healthy-lifestyle score. The inverse association between healthy-lifestyle score and stroke mortality did not vary by parental history of stroke in either sex, with the exception of paternal stroke history and stroke mortality in women. Overall, the respective hazard ratio (95% CI) for stroke for the highest healthy-lifestyle score (6–8) versus the worst score (0–3) category was 0.50 (0.35–0.71) with the presence of a maternal history of stroke and 0.47 (0.40–0.55) without such a history, 0.55 (0.40–0.76) with the presence of paternal history of stroke and 0.46 (0.38–0.54) without such a history, and 0.55 (0.43–0.72) with the presence of maternal and/or paternal history of stroke and 0.44 (0.36–0.53) without such a history. These results did not materially change when history of hypertension and diabetes mellitus were included in the multivariable model (model 2; not shown in table).

**Table 3.** Sex-specific multivariable hazard ratios (HRs) and 95% CIs for mortality from stroke, stratified by parental history of stroke

Parental history				Healthy-lifestyle score (points)				

				0–3	4	5	6–8	*P*-value for trend
**Men**								
	**Maternal**							
		**Positive** Person-years		15 075	9452	7628	7936	
			No.	58	34	19	19	
			Multivariable HR (95% CI)	1.00	0.87 (0.57–1.34)	0.57 (0.34–0.97)	0.58 (0.34–0.99)	0.01
		**Negative** Person-years		120 483	78 259	62 970	51 785	
			No.	293	158	115	78	
			Multivariable HR (95% CI)	1.00	0.77 (0.63–0.93)	0.65 (0.52–0.81)	0.52 (0.41–0.67)	<0.001
	**Paternal**							
		**Positive** Person-years		22 594	13 904	11 992	10 892	
			No.	76	38	21	23	
			Multivariable HR (95% CI)	1.00	0.75 (0.51–1.11)	0.43 (0.26–0.70)	0.52 (0.32–0.85)	0.001
		**Negative** Person-years		112 963	73 807	58 606	48 829	
			No.	275	154	113	74	
			Multivariable HR (95% CI)	1.00	0.79 (0.65–0.97)	0.70 (0.56–0.88)	0.53 (0.41–0.69)	<0.001
	**Paternal and/or maternal**							
		**Positive** Person-years		32 028	20 307	17 074	16 215	
			No.	106	64	36	38	
			Multivariable HR (95% CI)	1.00	0.90 (0.66–1.23)	0.55 (0.37–0.81)	0.61 (0.42–0.89)	0.001
		**Negative** Person-years		103 529	67 404	53 525	43 506	
			No.	245	128	98	59	
			Multivariable HR (95% CI)	1.00	0.74 (0.59–0.91)	0.67 (0.53–0.86)	0.49 (0.37–0.66)	<0.001
**Women**								
	**Maternal**							
		**Positive** Person-years		6879	11 602	15 198	22 547	
			No.	26	33	29	37	
			Multivariable HR (95% CI)	1.00	0.85 (0.50–1.43)	0.58 (0.34–1.00)	0.58 (0.34–0.98)	0.03
		**Negative** Person-years		56 220	91 578	121 010	177 136	
			No.	141	146	161	155	
			Multivariable HR (95% CI)	1.00	0.79 (0.62–0.99)	0.79 (0.63–1.00)	0.65 (0.51–0.82)	0.001
	**Paternal**							
		**Positive** Person-years		10 575	17 183	21 096	32 243	
			No.	23	41	37	47	
			Multivariable HR (95% CI)	1.00	1.22 (0.73–2.04)	1.04 (0.61–1.77)	1.00 (0.59–1.69)	0.76
		**Negative** Person-years		52 523	85 998	115 112	167 440	
			No.	144	138	153	145	
			Multivariable HR (95% CI)	1.00	0.73 (0.57–0.92)	0.71 (0.57–0.90)	0.57 (0.45–0.73)	<0.001
	**Paternal and/or maternal**							
		**Positive** Person-years		14 811	24 574	31 392	48 044	
			No.	40	59	53	69	
			Multivariable HR (95% CI)	1.00	1.03 (0.68–1.54)	0.81 (0.53–1.23)	0.80 (0.53–1.21)	0.19
		**Negative** Person-years		48 287	78 606	104 817	151 639	
			No.	127	120	137	123	
			Multivariable HR (95% CI)	1.00	0.72 (0.56–0.93)	0.74 (0.58–0.95)	0.58 (0.44–0.75)	0.001
**Total**								
	**Maternal**							
		**Positive** Person-years		21 953	21 054	22 826	30 493	
			No.	84	67	48	56	
			Multivariable HR (95% CI)	1.00	0.79 (0.57–1.09)	0.52 (0.37–0.75)	0.50 (0.35–0.71)	<0.001
		**Negative** Person-years		176 702	169 837	183 981	228 921	
			No.	434	304	276	233	
			Multivariable HR (95% CI)	1.00	0.70 (0.60–0.81)	0.62 (0.53–0.72)	0.47 (0.40–0.55)	<0.001
	**Paternal**							
		**Positive** Person-years		33 169	31 087	33 088	43 135	
			No.	99	79	58	70	
			Multivariable HR (95% CI)	1.00	0.78 (0.58–1.06)	0.55 (0.40–0.77)	0.55 (0.40–0.76)	<0.001
		**Negative** Person-years		165 486	159 805	173 719	216 269	
			No.	419	292	266	219	
			Multivariable HR (95% CI)	1.00	0.70 (0.60–0.81)	0.62 (0.53–0.72)	0.46 (0.38–0.54)	<0.001
	**Paternal and/or maternal**							
		**Positive** Person-years		46 839	44 881	48 466	64 258	
			No.	146	123	89	107	
			Multivariable HR (95% CI)	1.00	0.83 (0.65–1.06)	0.57 (0.43–0.74)	0.55 (0.43–0.72)	<0.001
		**Negative** Person-years		151 816	146 010	158 342	195 146	
			No.	372	248	235	182	
			Multivariable HR (95% CI)	1.00	0.67 (0.57–0.79)	0.62 (0.52–0.73)	0.44 (0.36–0.53)	<0.001

Multivariable adjustment: age, sex (for all subjects), perceived mental stress, educational level, and regular employment.

We examined age-specific associations between healthy-lifestyle score and mortality from stroke, stratified by parental history of stroke (Table 4). The inverse associations between healthy-lifestyle score and stroke mortality were stronger in the younger age group than in the older one, but they did not vary by parental history of stroke in either age group.

**Table 4.** Age-specific multivariable hazard ratios (HRs) and 95% CIs for mortality from stroke, stratified by parental history of stroke

Parental history				Healthy-lifestyle score (points)				

				0–3	4	5	6–8	*P*-value for trend
**Age 40–59 years**								
	**Maternal**							
		**Positive**		11 833	11 257	11 864	16 701	
			No.	18	11	5	8	
			Multivariable HR (95% CI)	1.00	0.63 (0.30–1.36)	0.27 (0.10–0.74)	0.30 (0.13–0.72)	0.002
		**Negative** Person-years		114 253	108 263	117 929	154 643	
			No.	84	54	48	45	
			Multivariable HR (95% CI)	1.00	0.65 (0.46–0.92)	0.54 (0.37–0.78)	0.38 (0.26–0.55)	<0.001
	**Paternal**							
		**Positive** Person-years		19 497	17 564	18 863	26 583	
			No.	27	13	8	19	
			Multivariable HR (95% CI)	1.00	0.49 (0.25–0.95)	0.28 (0.12–0.62)	0.43 (0.23–0.81)	0.005
		**Negative** Person-years		106 590	101 957	110 931	144 760	
			No.	75	52	45	34	
			Multivariable HR (95% CI)	1.00	0.71 (0.50–1.01)	0.58 (0.39–0.84)	0.33 (0.22–0.50)	<0.001
	**Paternal and/or maternal**							
		**Positive** Person-years		26 996	25 521	26 809	38 207	
			No.	39	22	10	21	
			Multivariable HR (95% CI)	1.00	0.56 (0.33–0.95)	0.24 (0.12–0.49)	0.34 (0.19–0.59)	<0.001
		**Negative** Person-years		99 090	94 000	102 984	133 136	
			No.	63	43	43	32	
			Multivariable HR (95% CI)	1.00	0.70 (0.48–1.04)	0.66 (0.44–0.97)	0.37 (0.24–0.58)	<0.001
**Age 60–79 years**								
	**Maternal**							
		**Positive** Person-years		10 120	9797	10 962	13 782	
			No.	66	56	43	48	
			Multivariable HR (95% CI)	1.00	0.84 (0.58–1.20)	0.59 (0.40–0.88)	0.56 (0.38–0.83)	0.001
		**Negative** Person-years		62 449	61 574	66 052	74 279	
			No.	350	250	228	188	
			Multivariable HR (95% CI)	1.00	0.71 (0.60–0.84)	0.63 (0.54–0.75)	0.49 (0.41–0.59)	<0.001
	**Paternal**							
		**Positive** Person-years		13 673	13 523	14 226	16 553	
			No.	72	66	50	51	
			Multivariable HR (95% CI)	1.00	0.89 (0.64–1.25)	0.65 (0.45–0.95)	0.58 (0.40–0.85)	0.002
		**Negative** Person-years		58 897	57 848	62 788	71 509	
			No.	344	240	221	185	
			Multivariable HR (95% CI)	1.00	0.70 (0.59–0.82)	0.63 (0.53–0.74)	0.49 (0.41–0.59)	<0.001
	**Paternal and/or maternal**							
		**Positive** Person-years		19 843	19 360	21 657	26 051	
			No.	107	101	79	86	
			Multivariable HR (95% CI)	1.00	0.93 (0.71–1.22)	0.69 (0.51–0.93)	0.64 (0.48–0.86)	0.001
		**Negative** Person-years		52 726	52 010	55 357	62 010	
			No.	309	205	192	150	
			Multivariable HR (95% CI)	1.00	0.66 (0.55–0.79)	0.61 (0.51–0.73)	0.46 (0.37–0.56)	<0.001

Multivariable adjustment: age, sex (for all subjects), perceived mental stress, educational level, and regular employment.

The Figure shows the multivariable hazard ratios of stroke mortality for each healthy-lifestyle score category among men and women with and without a parental history of stroke. Participants with a healthy-lifestyle score of 0 to 3 (worst score) and a maternal and/or paternal history of stroke were used as the reference. Among participants with and without a parental history of stroke, the hazard ratio decreased as healthy-lifestyle score increased. The hazard ratio for stroke mortality was generally higher among participants with a parental history of stroke than in those without such a history. The trends were virtually identical in men and women.

**Figure.**
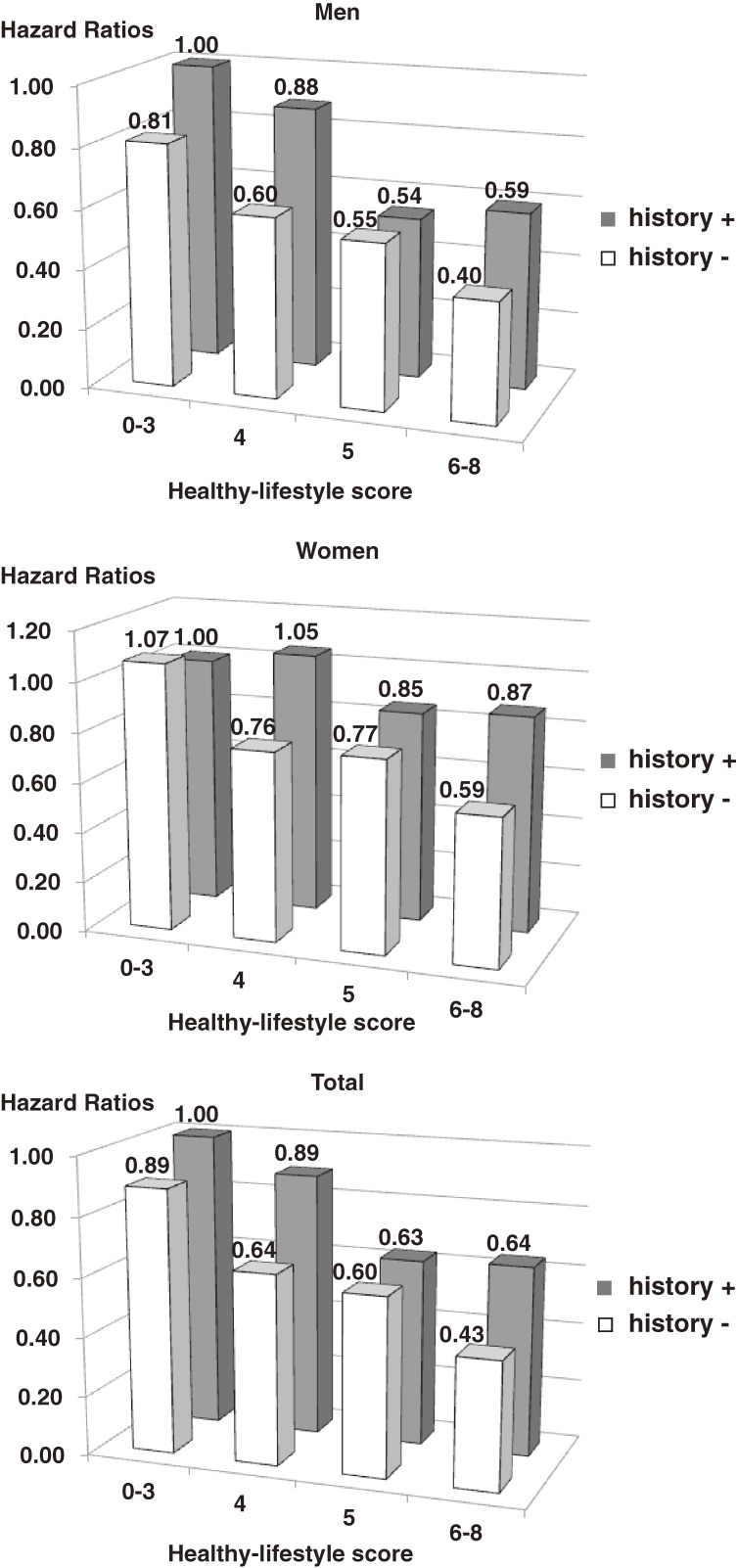
Multivariable hazard ratios for stroke mortality for each healthy-lifestyle score category for men, women, and all participants with and without a parental history of stroke, using those with a healthy-lifestyle score of 0–3 and a maternal and/or paternal history of stroke as the reference category.

## DISCUSSION

In a prospective large cohort study of 53 691 Japanese men and women aged 40 to 79 years, we observed a 16% to 40% increase in stroke mortality risk in participants who had a parental history of stroke. We also found that the inverse relationship observed between healthy lifestyle behaviors and mortality was similar in both men and women and stronger in younger than in older adults, regardless of parental history of stroke, with the exception of paternal stroke history and stroke mortality in women.

A number of previous studies reported an association between parental history and risk of cardiovascular disease and stroke in offspring. In the Framingham Offspring Study, which followed 2302 men and women (28–62 years) for up to 8 years, Lloyd-Jones et al reported multivariate odds ratios (95% CI) for cardiovascular disease of 2.0 (1.2–3.1) in men and 1.7 (0.9–3.1) in women, among participants with at least 1 parent with early-onset cardiovascular disease (onset age <55 years in father, <65 years in mother), and 1.5 (0.9–2.4) in men and 1.1 (0.6–2.1) in women, among those at least 1 parent with non–early-onset cardiovascular disease, including ischemic stroke.^7^ In addition, on the basis of data from 149 896 person-years that were obtained from 14 371 Finnish men and women aged 25 to 64 years, the multivariable hazard ratio (95% CIs) for stroke associated with any parental history of stroke was 1.9 (1.2–2.9) in men and 1.8 (1.2–2.8) in women.^3^ Another study of 13 775 individuals aged 45 to 64 years from the atherosclerosis risk in communities (ARIC) cohort reported a multivariable odds ratio (95% CI) for subclinical stroke (cerebral infarct >3 mm) of 1.6 (1.2–2.2) in men and women combined; however, the multivariable hazard ratio for developing clinical stroke was 1.1 (0.8–1.4), which was not significant.^4^ Kadota et al showed no association between parental history of stroke and stroke mortality in a 19-year follow-up study of 8 037 randomly selected Japanese adults (age, ≥30 years) from the general population of NIPPON DATA 80.^5^

Our study is the first to show a positive association between parental history of stroke and stroke-related mortality among their offspring in a Japanese population. As compared with 2 previous studies that examined this association,^3^^,^^4^ the magnitude of the present association is smaller. One reason for this discrepancy is the difference in respective outcomes, ie, incidence and mortality, between the previous studies and the present study. Stroke mortality may be affected by survival factors, which can dilute associations between parental history and disease. In addition, the association of parental history of stroke and offspring stroke mortality was stronger among younger persons than older ones, which was consistent with the findings of the Framingham heart study.^7^

The hazard ratios of stroke mortality associated with parental history of stroke became smaller when we added history of hypertension and history of diabetes to the model. This finding supports the hypothesis that hypertension and diabetes mellitus are partial mediators of the association between parental history of stroke and stroke mortality in offspring.

The association between maternal stroke history and stroke mortality tended to be stronger than the association between paternal stroke history and stroke mortality in both men and women, although the difference was not statistically significant. As compared with fathers, mothers are believed to share a greater number of lifestyle behaviors with their offspring, and that might be a reason for our result.

In the present study, the impact of parental history on stroke mortality was much lower than that of lifestyle behaviors. The PAF for stroke mortality for maternal and/or paternal history of stroke was 5.4% in men and 4.3% in women. In our previous study, the PAF for stroke mortality in adults who did not have 7 to 8 healthy lifestyle behaviors was 45.0% in men and 43.4% in women.^13^

The effect modification of parental history of stroke on the association between healthy lifestyle behaviors and stroke mortality has not been studied. Our study showed that the association of healthy lifestyle behaviors and stroke mortality was similar in men and women and that younger persons had a stronger inverse association than did older ones. Neither of the associations was influenced by parental history of stroke. There was, however, an exception to this result, ie, a lack of a significant association between healthy-lifestyle score and stroke mortality in women with a paternal stroke history. The reasons for this discrepancy are unknown, but it might be a chance phenomenon due in part to the small number of participants in the reference group, namely, those with a paternal history of stroke and the worst healthy-lifestyle score (0–3).

The strengths of our study are as follows: (1) it was a large-scale, cohort-based study that enrolled participants from all over Japan, with over 1500 deaths reported during long-term follow-up, (2) multiple lifestyle variables were collected at baseline, and (3) adjustments were made for multiple potential confounding variables. These advantages allowed us to estimate the impact of parental history of stroke on stroke-related mortality in offspring, and further allowed us to assess the potential modifying effect of parental history of stroke on the association between healthy lifestyle behaviors and stroke mortality in offspring.

The study did have some limitations that warrant mention. First, we did not have information on the age at which parents had a stroke. The absence of this information meant we were unable to investigate the relationship between parental history of early-onset stroke and stroke-related mortality in offspring. Second, baseline data on lifestyle were only obtained at the initial assessment. It is possible that lifestyle changes occurred during the follow-up period. Consequently, nondifferential measurement errors would have attenuated the observed associations, when the actual/real associations might have been stronger. We used stroke mortality rather than stroke incidence as an endpoint; thus, stroke onset during follow-up might have induced lifestyle changes and consequently influenced mortality risk in some individuals. Finally, there might be a concern regarding selection bias due to the exclusion of 57 101 participants who consented to the study but lacked the necessary information to be included in the analysis. However, the exclusion of these individuals was unlikely to influence these relationships, as their baseline characteristics were similar to those of the rest of the cohort.

### Conclusions

In conclusion, a parental history of stroke was associated with an increased risk of stroke mortality among a Japanese population. The association tended to be stronger among study participants who had a maternal versus a paternal history of stroke. The inverse association between lifestyle behaviors and stroke mortality in offspring was similar in men and women and was stronger for younger persons than older ones, regardless of parental history of stroke. However, there was no significant association between healthy life scores and stroke mortality rates in women with a paternal stroke history. These results confirm the need to motivate individuals to modify their lifestyle to prevent stroke, particularly when their parents have a history of stroke.

## References

[1] Li R, Bensen JT, Hutchinson RG, Province MA, Hertz-Picciotto I, Sprafka JM, Family risk score of coronary heart disease (CHD) as a predictor of CHD: The atherosclerosis risk in communities (ARIC) study and the NHLBI family heart study. Genet Epidemiol. 2000;18:236–50 10.1002/(SICI)1098-2272(200003)18:3<236::AID-GEPI4>3.0.CO;2-0 10723108

[2] Ciruzzi M, Schargrodsky H, Rozlosnik J, Pramparo P, Delmonte H, Rudich V, Frequency of family history of acute myocardial infarction in patients with acute myocardial infarction. Argentine FRICAS (Factores de Riesgo Coronario en America del Sur) Investigators. Am J Cardiol. 1997;80:122–7 9230145 10.1016/s0002-9149(97)00304-4

[3] Jousilahti P, Rastenyte D, Tuomilehto J, Sarti C, Vartiainen E Parental history of cardiovascular disease and risk of stroke. A prospective follow-up of 14371 middle-aged men and women in Finland. Stroke. 1997;28:1361–6 10.1161/01.STR.28.7.1361 9227684

[4] Morrison AC, Fornage M, Liao D, Boerwinkle E Parental history of stroke predicts subclinical but not clinical stroke: The Atherosclerosis Risk in Communities Study. Stroke. 2000;31:2098–102 10.1161/01.STR.31.9.2098 10978036

[5] Kadota A, Okamura T, Hozawa A, Kadowaki T, Murakami Y, Hayakawa T, Relationships between family histories of stroke and of hypertension and stroke mortality: NIPPON DATA80, 1980–1999. Hypertens Res. 2008;31:1525–31 10.1291/hypres.31.1525 18971526

[6] Greenland P, Alpert JS, Beller GA, Benjamin EJ, Budoff MJ, Fayad ZA, 2010 ACCF/AHA guideline for assessment of cardiovascular risk in asymptomatic adults: A report of the American College of Cardiology Foundation/American Heart Association Task Force on Practice Guidelines. J Am Coll Cardiol. 2010;56:e50–103 10.1016/j.jacc.2010.09.001 21144964

[7] Lloyd-Jones DM, Nam BH, D’Agostino RB Sr, Levy D, Murabito JM, Wang TJ, Parental cardiovascular disease as a risk factor for cardiovascular disease in middle-aged adults: A prospective study of parents and offspring. JAMA. 2004;291:2204–11 10.1001/jama.291.18.2204 15138242

[8] Shih PA, O’Connor DT Hereditary determinants of human hypertension: Strategies in the setting of genetic complexity. Hypertension. 2008;51:1456–64 10.1161/HYPERTENSIONAHA.107.090480 18413494 PMC2587105

[9] Weiss LA, Pan L, Abney M, Ober C The sex-specific genetic architecture of quantitative traits in humans. Nat Genet. 2006;38:218–22 10.1038/ng1726 16429159

[10] Harrison TA, Hindorff LA, Kim H, Wines RC, Bowen DJ, McGrath BB, Family history of diabetes as a potential public health tool. Am J Prev Med. 2003;24:152–9 10.1016/S0749-3797(02)00588-3 12568821

[11] Coady SA, Jaquish CE, Fabsitz RR, Larson MG, Cupples LA, Myers RH Genetic variability of adult body mass index: A longitudinal assessment in framingham families. Obes Res. 2002;10:675–81 10.1038/oby.2002.91 12105290

[12] Munafò M, Clark T, Johnstone E, Murphy M, Walton R The genetic basis for smoking behavior: A systematic review and meta-analysis. Nicotine Tob Res. 2004;6:583–97 10.1080/14622200410001734030 15370155

[13] Eguchi E, Iso H, Tanabe N, Wada Y, Yatsuya H, Kikuchi S, Healthy lifestyle behaviors and cardiovascular mortality among Japanese men and women: The Japan collaborative cohort study. Eur Heart J. 2012;33:467–77 10.1093/eurheartj/ehr429 22334626

[14] Ohno Y, Tamakoshi A; JACC Study Group Japan collaborative cohort study for evaluation of cancer risk sponsored by monbusho (JACC study). J Epidemiol. 2001;11:144–50 10.2188/jea.11.144 11512570 PMC11735075

[15] Iwai N, Hisamichi S, Hayakawa N, Inaba Y, Nagaoka T, Sugimori H, Validity and reliability of single-item questions about physical activity. J Epidemiol. 2001;11:211–8 10.2188/jea.11.211 11579928 PMC11769784

[16] Date C, Fukui M, Yamamoto A, Wakai K, Ozeki A, Motohashi Y, Reproducibility and validity of a self-administered food frequency questionnaire used in the JACC study. J Epidemiol. 2005;15 Suppl 1:S9–23 10.2188/jea.15.S9 15881192 PMC8565864

[17] Tamakoshi A, Yoshimura T, Inaba Y, Ito Y, Watanabe Y, Fukuda K, Profile of the JACC study. J Epidemiol. 2005;15 Suppl 1:S4–8 10.2188/jea.15.S4 15881191 PMC8565873

[18] Miettinen OS Proportion of disease caused or prevented by a given exposure, trait or intervention. Am J Epidemiol. 1974;99:325–32 4825599 10.1093/oxfordjournals.aje.a121617

